# Intraoperative Management of a Distal Femoral Neck Cut Complicated by Greater Trochanter Avulsion During Total Hip Arthroplasty With High Offset Stem and Cement Fixation

**DOI:** 10.7759/cureus.89966

**Published:** 2025-08-13

**Authors:** Paul Gerges, Vincent Lee, Bryce Bossinger, Ricardo Rios, Ralph Rizk

**Affiliations:** 1 School of Medicine, Edward Via College of Osteopathic Medicine - Carolinas Campus, Spartanburg, USA; 2 School of Medicine, Edward Via College of Osteopathic Medicine - Auburn Campus, Auburn, USA; 3 Surgery, Edward Via College of Osteopathic Medicine - Virginia Campus, Blacksburg, USA; 4 Orthopedic Surgery, Ralph Rizk Orthopedics, Jacksonville, USA

**Keywords:** avulsion fracture, cement, surgical complication, total hip athroplasty, total hip replacement (thr)

## Abstract

Total hip replacement (THR) is a common orthopedic surgery performed to relieve pain and restore mobility in patients with severe hip arthritis. While most surgeries proceed without major issues, rare complications during the operation can make the procedure more challenging and potentially affect recovery. We present the case of a 77-year-old man who underwent a right THR through the anterior surgical approach to treat advanced osteoarthritis. During surgery, two unexpected problems occurred: the cut made to remove the damaged femoral head was placed too far down the bone, and a portion of the upper thigh bone called the greater trochanter, where important muscles attach, became detached. Before surgery, detailed planning was done using CT-based Mako robotic navigation, which helped choose the most suitable implant and determine the correct positioning. During the procedure, a high-offset cemented femoral stem was selected to restore the patient’s natural hip mechanics. The detached greater trochanter was repaired using strong sutures passed through holes in the bone (transosseous fixation). After surgery, the patient was kept off the operated leg initially to protect the repair. He gradually progressed to partial weight-bearing by three months, and follow-up imaging showed excellent healing and implant position. Functionally, he returned to daily activities without pain. This case underscores the importance of adaptability during surgery, even with advanced planning. It also highlights how robotic planning, specialized implant selection, and careful repair of soft tissue attachments can be successfully combined to address unexpected challenges and achieve an excellent outcome.

## Introduction

Total hip arthroplasty (THA) is a reliable and effective procedure for managing end-stage osteoarthritis and other degenerative conditions of the hip. Key goals of THA include restoring joint biomechanics, equalizing leg length, and preserving soft tissue balance to optimize postoperative function. In recent years, technological advances such as CT-based robotic navigation have improved surgical accuracy and enabled surgeons to plan and execute complex reconstructions with greater confidence [1].

Despite these advances, intraoperative complications remain a challenge. High-offset femoral stems, cemented fixation, and transosseous sutures are valuable techniques for addressing these challenges [2-3]. Errors in femoral neck osteotomy or greater trochanter avulsion may compromise joint stability, offset, and muscle tensioning, particularly in anterior-approach THA, where exposure may be limited. Greater trochanteric injuries, although relatively uncommon, are associated with poorer abductor function and higher rates of dislocation [4]. Similarly, improper femoral neck cuts can reduce available bone stock for stem fixation and compromise leg length restoration [5].

This case report illustrates the intraoperative management of a 77-year-old male who experienced a distal femoral neck cut and greater trochanter avulsion during primary THA. 

## Case presentation

A 77-year-old male presented with advanced osteoarthritis of the right hip, characterized by chronic pain, stiffness, and significantly reduced mobility. The patient reported progressive difficulty performing activities of daily living, including walking, climbing stairs, and standing for prolonged periods. Conservative measures, including physical therapy, nonsteroidal anti-inflammatory drugs (NSAIDs), and intra-articular corticosteroid injections, had failed to provide lasting relief. The patient met the typical criteria for elective total hip replacement (THR) to alleviate pain and restore function, including adequate bone stock, preserved muscle function, no active infection, absence of severe cardiovascular risk, and a baseline Harris Hip Score indicating substantial functional impairment.

CT-based Mako robotic navigation (Stryker) was used for preoperative planning to optimize surgical precision. This technology allowed for detailed assessment of the patient’s hip anatomy, simulation of implant placement, and biomechanical optimization. The surgical plan included an anterior approach THR with a standard femoral neck cut at the anatomical neck, the use of a Trident® II Tritanium® acetabular cup (Stryker), and an Insignia cementless femoral stem (Stryker). A cementless femoral stem was initially selected based on the patient’s adequate proximal femoral bone stock and favorable bone quality on preoperative imaging, which were expected to allow reliable biologic fixation. This plan was consistent with standard practice for patients meeting these criteria and was supported by the CT-based preoperative assessment. Preoperative planning also accounted for potential challenges, including bone quality and soft tissue balance, to ensure readiness for intraoperative complications.

**Figure 1:**
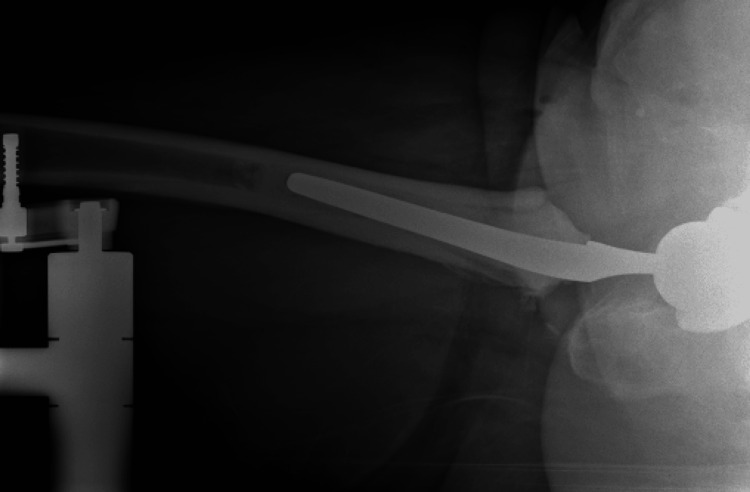
Intraoperative lateral radiograph showing the distal femoral neck cut Intraoperative lateral radiograph showing an overly distal femoral neck cut with minimal remaining neck, necessitating the use of a high-offset stem for biomechanical restoration.

**Figure 2:**
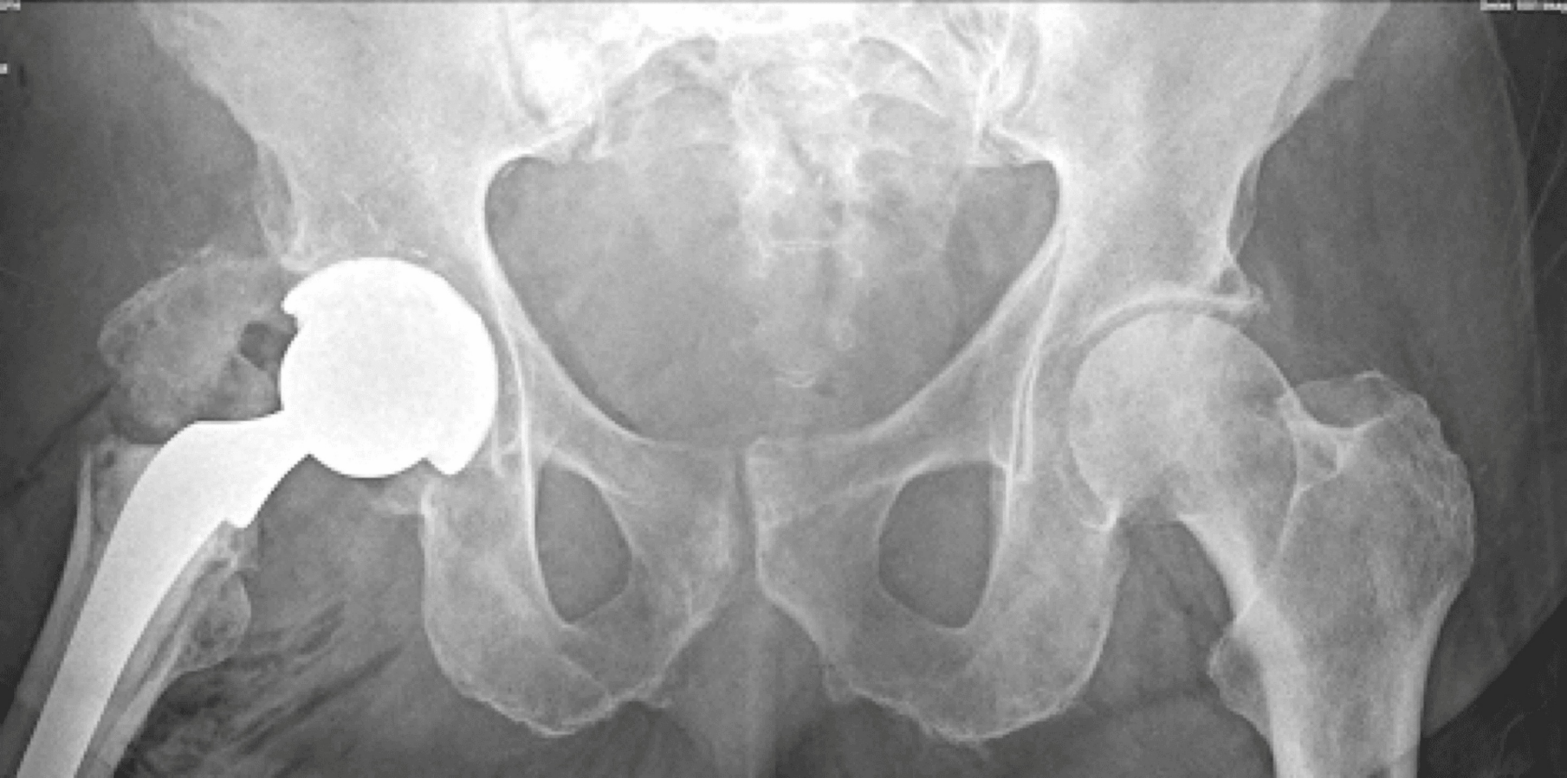
Postoperative anteroposterior radiograph showing intraoperative greater trochanter avulsion. Postoperative AP radiograph showing a displaced greater trochanter fragment resulting from intraoperative avulsion, adjacent to a well-positioned cemented femoral stem.

During the femoral neck osteotomy, an atypical, overly distal cut was made, compounded by an avulsion of the tip of the greater trochanter. The distal femoral neck cut and the greater trochanter avulsion are shown in Figures 1-2, respectively. This combination significantly compromised the remaining femoral neck length, creating challenges in restoring proper leg length, joint stability, and soft tissue tensioning. To address these complications, Mako robotic navigation was utilized to refine implant positioning and ensure optimal biomechanical restoration.

Following the distal femoral neck cut and greater trochanter avulsion, the femoral anatomy was carefully reassessed intraoperatively. It was determined that a high femoral offset was required to restore the patient’s hip biomechanics. The avulsed greater trochanter posed a particular challenge to maintaining soft tissue balance, especially for the abductor muscles, which are critical for stability and function postoperatively [6]. The use of preoperative CT-based planning and intraoperative navigation helped the surgical team identify the required modifications to achieve appropriate leg length and offset.

The surgical team selected a high-offset cemented femoral stem, the Accolade® C (Stryker), which features a 127° neck angle, to provide greater lateralization of the femur and optimize soft tissue balance. This adjustment effectively compensated for the compromised femoral neck length and greater trochanter avulsion, allowing for accurate leg length restoration while minimizing the risk of postoperative dislocation [7]. Accurate restoration of leg length is critical, as improper adjustments, whether shortening or over-lengthening, are known to negatively impact postoperative functional outcomes [2,7].

To manage the avulsion of the greater trochanter, transosseous sutures were employed to reattach the fragment. This technique stabilized the trochanter and restored the tension of the abductor musculature, ensuring optimal postoperative function. Repair of the greater trochanter was critical to maintaining abductor muscle strength and minimizing the risk of dislocation [8]. The use of transosseous sutures allowed for a robust fixation, essential for the patient’s successful recovery.

Due to the altered anatomy resulting from the distal femoral neck cut and greater trochanter avulsion, the intraoperative bone stock was found to be insufficient to support a press-fit cementless stem as originally planned. To achieve immediate stable fixation, a high-offset cemented Accolade® C stem (Stryker) was implanted using Simplex P high-viscosity bone cement (Stryker). The femoral canal was irrigated and dried, a distal plug was inserted, and the cement was applied retrograde under pressure. The stem was inserted, followed by a second pressurization to create a uniform cement mantle. A stem centralizer ensured proper alignment within the mantle, providing long-term stability [3,8]. A stem centralizer was used to ensure proper alignment of the stem within the cement mantle, providing long-term stability.

The acetabular component, a Trident® II Tritanium® Clusterhole Acetabular Shell, was robotically placed using Mako navigation. This 3D-printed cup was designed to mimic cancellous bone without requiring additional coating. The cup was positioned at 40° inclination and 20° anteversion to optimize biomechanics and reduce the risk of dislocation. Virtual range-of-motion testing was performed intraoperatively using the Mako system, confirming that the cup placement would not cause impingement [9].

Following implantation of the femoral and acetabular components, the hip joint was reduced, and stability was assessed. The high-offset stem successfully restored proper leg length and offset, while the transosseous suture technique for the greater trochanter avulsion ensured proper soft tissue tensioning. The hip demonstrated excellent stability with no signs of impingement. Range of motion testing confirmed the absence of subluxation or dislocation [2,6].

The patient progressed as expected, with no signs of dislocation or infection. Physical therapy supported non-weight-bearing recovery due to the trochanteric fracture and abductor repair. A manual wheelchair was prescribed, justified by the patient’s inability to participate in mobility-related activities of daily living (MRADLs) with a cane or walker. Despite mobility limitations, the patient demonstrated sufficient upper body strength and cognitive function to self-propel. At the two-week postoperative visit, the patient demonstrated satisfactory wound healing with no signs of infection. The pain was well-controlled with oral analgesics, and the patient remained non-weight bearing on the right lower extremity as advised. Physical therapy focused on upper body strengthening and maintaining mobility with a manual wheelchair. Neurovascular examination showed no deficits, and the greater trochanter repair remained stable. The patient reported adherence to the rehabilitation protocol and expressed motivation to continue therapy. 

**Figure 3:**
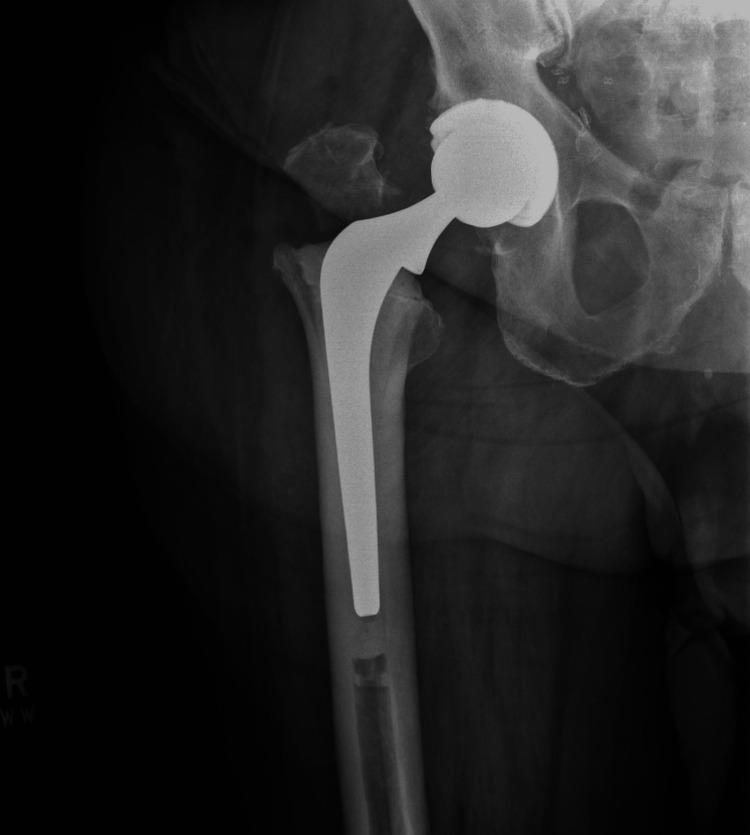
Postoperative cross-table lateral radiograph of the right hip Postoperative lateral radiograph confirming proper alignment of the cemented femoral stem and acetabular component without signs of dislocation or loosening.

By the one-month mark, the patient had shown significant progress in upper body mobility and functional movement within the constraints of non-weight-bearing status. The manual wheelchair remained the primary mode of mobility, but the patient was becoming increasingly independent in transfers and other activities of daily living. The surgical site was fully healed, and the postoperative imaging in Figure 3 confirmed proper implant positioning, with no signs of loosening or subsidence. Physical therapy introduced gentle range-of-motion exercises for the right hip, maintaining precautions to protect the repaired abductor mechanism.

At three months, the patient had transitioned to partial weight bearing under close supervision. Functional movement and mobility had improved significantly, with the patient demonstrating increased strength in the right lower extremity. Gait training began with assistive devices, and physical therapy sessions emphasized gradual weight-bearing progression and abductor strengthening. Radiographic evaluation confirmed implant stability and a healed greater trochanter repair. The patient reported minimal pain and significant improvements in daily function, including the ability to engage in light household activities with minimal assistance.

## Discussion

Intraoperative challenges during THR, such as distal femoral neck cuts and greater trochanter avulsion, require tailored solutions to restore hip biomechanics. In this case, robotic navigation with Mako played a crucial role in achieving precise cup placement and avoiding impingement. Robotic navigation technology has proven to achieve more precise interoperative component positioning, which has proven clinical benefits by resulting in decreased complications [10]. The use of a high-offset femoral stem (Accolade® C) effectively restored lateralization and leg length, compensating for the compromised neck length. High-viscosity cement provided immediate fixation, reducing the risk of micromotion or implant loosening. A distal spacer and stem centralizer further enhanced alignment within the cement mantle.

The 3D-printed Trident® II acetabular component facilitated optimal integration, mimicking cancellous bone structure without requiring additional coating. Transosseous sutures for the greater trochanter avulsion successfully re-tensioned the abductor muscles, maintaining their function and reducing dislocation risk. Literature supports these techniques as effective for managing complex intraoperative complications, emphasizing the importance of adaptability during surgery [3,6,8].

Similar intraoperative complications have been described in the literature, particularly involving greater trochanter fractures during or after a THA. These injuries are associated with impaired abductor strength, increased risk of dislocation, and poorer postoperative outcomes, including reduced functional scores and greater patient-reported pain [11]. While some cases may be managed conservatively, displaced or symptomatic avulsions often require surgical repair to restore stability and soft tissue balance. However, few reports detail a combined approach using robotic navigation, high-offset cemented stems, and transosseous suture fixation. To our knowledge, no previously published case has described the simultaneous occurrence of both a greater trochanter avulsion and an overly distal femoral neck cut managed with this combination of techniques. This case demonstrates how an integrated strategy can address multiple concurrent intraoperative complications and support favorable biomechanical restoration and clinical recovery.

## Conclusions

An atypical distal femoral neck cut and greater trochanter avulsion during THR can significantly complicate the restoration of hip biomechanics. The use of a high-offset femoral stem, cement fixation, and transosseous sutures for the avulsed greater trochanter provided a successful solution in this case. Surgeons must be adaptable and consider these techniques when managing complex intraoperative challenges to ensure optimal patient outcomes.
